# Experimental Study on Chloride Ion Diffusion in Concrete under Uniaxial and Biaxial Sustained Stress

**DOI:** 10.3390/ma13245717

**Published:** 2020-12-15

**Authors:** Xiaokang Cheng, Jianxin Peng, C.S. Cai, Jianren Zhang

**Affiliations:** 1 School of Civil Engineering, Changsha University of Science & Technology, Changsha 410114, China; chengxiaokang1994@163.com (X.C.); jianrenz@hotmail.com (J.Z.); 2 Department of Civil & Environmental Engineering, Louisiana State University, Baton Rouge, LA 70803, USA; cscai@lsu.edu

**Keywords:** concrete, uniaxial/biaxial sustained compressive stress, chloride concentration, chloride diffusion coefficient, stress level

## Abstract

The existence of axial and lateral compressive stress affect the diffusion of chloride ions in concrete will lead to the performance degradation of concrete structure. This paper experimentally studied the chloride diffusivity properties of uniaxial and biaxial sustained compressive stress under one-dimensional chloride solution erosion. The influence of different sustained compressive stress states on chloride ion diffusivity is evaluated by testing chloride concentration in concrete. The experiment results show that the existence of sustained compressive stress does not always inhibit the diffusion of chloride ions in concrete, and the numerical value of sustained compressive stress level can affect the diffusion law of chloride ions in concrete. It is found that the chloride concentration decreases most when the lateral compressive stress level is close to 0.15 times the compressive strength of concrete. In addition, the sustained compressive stress has a significant effect on chloride ion diffusion of concrete with high water/cement ratio. Then, the chloride diffusion coefficient model under uniaxial and biaxial sustained compressive stress is established based on the apparent chloride diffusion coefficient. Finally, the results demonstrate that the chloride diffusion coefficient model is reasonable and feasible by comparing the experimental data in the opening literature with the calculated values from the developed model.

## 1. Introduction

The durability of concrete material is considered as one of the most important problems of concrete structures. Chloride corrosion is the main factor of concrete material performance degradation [1,2,3,4]. The external load affects the transport of chloride ions in the concrete cover. It changes the transport rate of chloride ions to the steel bars surface and the corrosion initiation time [5,6,7,8]. Therefore, the influence of load on chloride diffusion in concrete be fully regarded. Some scholars have developed to study the law of chloride transportion in concrete under stress. Hani R. Samaha and Kenneth C. Hover [9] found that the migration rate of chloride ions in concrete structure is accelerated after the stress of 75% compressive strength is applied to concrete. However, the change of chloride ion migration rate is not obvious as if the applied stress is less than 75% compressive strength. The above study has obtained some conclusions about chloride ions transport in concrete for they were based on the state after stress damage. Most concrete structures are subjected to sustained stress during use, and chloride ion transmission characteristics are related to the increase of stress and time [10]. Due to the concrete materials elasticity, some microcracks in concrete can be recovered after unloading. The test results show that the diffusion law of chloride ions do not change obviously after stress damage of concrete [11,12,13,14].

Some studies have described the performance of chloride diffusion under sustained load. Wang et al. [10] conducted that the increase of sustained compressive stress will not always hinder the diffusion of chloride ions. The experimental results show that the chloride diffusion coefficient increases when the sustained compressive stress is higher than 0.3*f_c_* (e.g., *f_c_* = prism compressive strength at 28 days). However, chloride ions diffusion in concrete will also be affected by biaxial sustained stress. Xingcai Wei [15] studied chloride ions permeability experiments using admixture concrete under biaxial sustained pressure stress, and found that the increase of compressive stress does not always inhibit the chloride ion diffusion. The relationship between stress level and chloride concentration of concrete with different *w/c* (e.g., water/cement) ratios has not been fully investigated in the above studies. Few studies focus on chloride diffusion properties of different *w/c* concrete under biaxial sustained compressive stress. Therefore, it is necessary to investigate the chloride diffusion of concrete structures with different *w/c* ratios under biaxial sustained compressive stress.In view of the preceding brief discussion, this study is to analyze the diffusion law of chloride ions in concrete under different compressive stress conditions. In this paper, a series of chloride ion penetration experiments of concrete under uniaxial and biaxial sustained compressive stress are carried out, and the variation law of chloride concentration in concrete is analyzed. Then, the apparent chloride diffusion coefficient under stress state is obtained by Fick’s second law diffusion equation, and the change of apparent chloride diffusion coefficient under uniaxial and biaxial sustained compressive stress is analyzed. The chloride diffusion models under different stress states are derived. The applicability of the diffusion models is verified by the experimental data from the published literatures.

## 2. Experimental Program

### 2.1. Materials and Specimen Preparation

The materials requirements of concrete were as follows. Concrete specimens were divided into three categories, namely, categories D, categories E and categories F. Among them, Categories D and E were used to study the influence of uniaxial sustained and biaxial sustained compressive load on chloride intrusion, respectively. Categories F were subjected to non-loading. The Portland cement had a characteristic compressive strength of 42.5 MPa supplied by the Changsha and conforming to the GB 175-2007 standard [16]. The chemical composition of the cement is shown in Table 1. Gravel with maximum size of 20 mm and density of 1550 kg/m^3^ available in Liu yang City, Hunan Province in China is used as coarse aggregate. The natural river sand was used as fine aggregate with fineness modulus ranging from 2.3 to 3.1. All concrete specimens were added with poly carboxylic ethers superplasticizer (SP) produced by Shandong Huawei Yinkai Building Materials Science and Technology Co., Ltd. The concrete mix ratio is shown in Table 2. After these specimens were formed, they were cured in a curing room at a temperature of 20 ± 2 °C and at a relative humidity of 90 ± 5% for 28 days. Some concrete specimens were tested for compressive strength, and the measured values of compressive strength of concrete with *w/c* ratio of 0.44, 0.40 and 0.36 were 47.21 MPa, 52.68 MPa and 56.78 MPa, respectively. The remaining concrete specimens were subjected to sustained compressive stress in the chloride ion diffusion experiment.

### 2.2. Experimental and Test Arrangement

**Figure 1:**
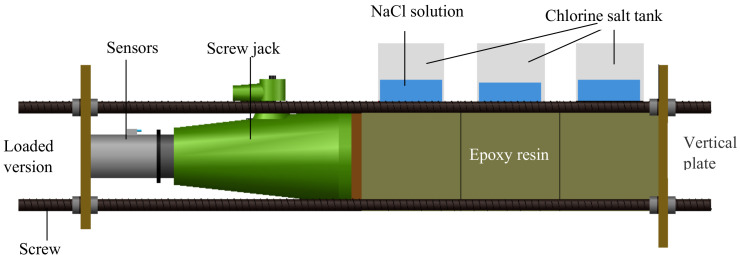
Uniaxial loading.

**Figure 2:**
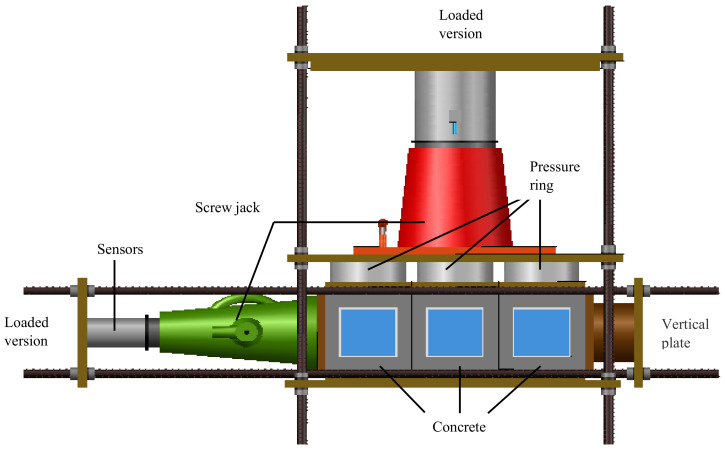
Biaxial loading.

In order to analyze the one-dimensional diffusion law of chloride ions in concrete under sustained pressure stress, the other five surfaces of the specimens were coated with epoxy resin except the penetration surface. The 100 mm central area of the concrete specimen’s upper surface was taken as the chloride ions penetration surface to reduce the influence of concrete boundary on the chloride ion diffusion process, and the upper surface was treated with wear reduction before the concrete specimens with different w/c ratios were placed into the loading device for loading test. The stress on the long side and short side of the concrete specimen was expressed as *σ_cx_* and *σ_cy_*, respectively. For categories D (one dimensional loading), the concrete specimens are put neatly in the axial direction of the loading device, and the compressive stress was monitored and controlled by the pressure readings of the screw jack, as shown in Figure 1. For categories E (two-dimensional loading), except that the axial direction is consistent with that of categories D, each concrete specimen is provided with a force transfer plate on the lateral side as shown in Figure 2. A compressive ring with the same size as the side length of the concrete specimen is placed on the force transfer plate. One side of the loading plate was in contact with the screw jack, and the other side was placed on the pressure ring. According to adjusting the screw jack to control the numerical values of the pressure rings, the numerical values of the three pressure rings were equal and reach the stress design value. Some scholars have found that when the biaxial compressive stress ratio *σ_cx_*/*σ_cy_* = 1:1–1:0.25, and the properties of concrete materials change greatly [17,18]. Categories F, the control group of concrete specimens, was not subjected to stress. The concrete with *w/c* ratio of 0.44, 0.4 and 0.36 was expressed by parameters Fd0, Fe0 and Ff0, respectively. The compressive stress applied to concrete specimens in the same loading device was the same. The stress state details of all specimens with their definitions are listed in Table 3 and Table 4.

### 2.3. Diffusion Experiment and Determination of Chloride Concentration

After the compressive stress was stabilized, the prefabricated chloride trough was placed on the concrete permeable surface, and the chloride trough was adhered to the concrete specimens by the adhesive. The 3.5% NaCl solution was inverted in the chloride trough with a beaker. Chloride ions penetration experiment was carried out in artificial climate simulation box, and the temperature was set between 20–22 °C. The exposure time of concrete specimens to chlorine salt was 2 months. The solution in the chlorine salt trough was replaced every week to ensure the solution did not precipitate and the chloride concentration was stable. After that, the sampling of concrete specimens shall be carried out according to the JTS/T 236-2019 standard [19]. The drilling rig was vertical to the concrete permeated surface. Six measuring points were obtained by drilling holes inward to collect powder, which had the distance of 0–5 mm, 5–10 mm, 10–15 mm, 15–20 mm, 20–25 mm and 25–30 mm from the permeated surface. To avoid the influence of errors, three data were taken for the drilling depth of the same layer. The powders were dried by an oven at a temperature 105 °C for 2 hours, and then cooled down in a desiccator to the room temperature. After that, the chloride concentration of concrete powder sample was also measured in compliance with the standard JTS/T 236-2019. The stress level *μ* can be used to describe the distribution of chloride ion diffusion caused by sustained stress [20,21,22,23,24].

(1) $$\mu_{1,2}=\frac{\sigma_{c(x,y)}}{f_{c}}$$

where *f_c_* is the measured compressive strength of concrete. *σ_c_* is the applied compressive stress value, in which the stress *σ_cx_* and *σ_cy_* correspond *μ_1_* and *μ_2_*, respectively.

## 3. Test Results and Discussion

### 3.1. Distribution of Chloride Concentration under Different Axial Compressive Stresses

**Figure 3:**
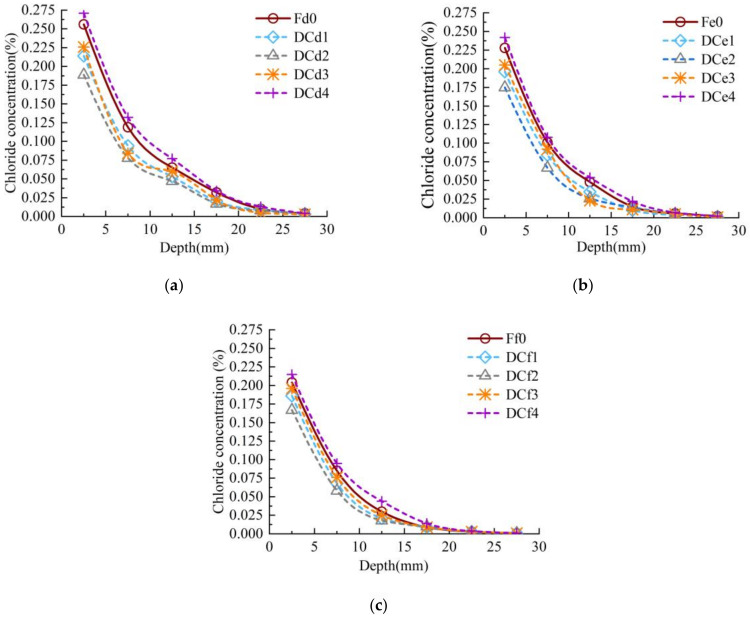
Chloride concentration distribution of concrete with three *w/c* ratios under uniaxial sustained stress: (**a**) *w/c* = 0.44; (**b**) *w/c* = 0.4; (**c**) *w/c* = 0.36.

The chloride concentration distributions of the concrete specimens under uniaxial sustained compressive stress (i.e., categories D) and zero stress (i.e., categories F) are shown in Figure 3. Take the chloride concentration values at a depth of 7.5mm as an example. The chloride concentration values of DCd1, DCd2, DCd3 and DCd4 specimens are 21.05%, 34.58%, 29.41% and −10.85% lower than those of Fd0 specimens in the same layer as seen in Figure 3a. It can be shown from Figure 3b that the chloride concentrations values of DCe1, DCe2, DCe3 and DCe4 specimens are 16.83%, 33.75%, 27.84% and −6.92% lower than those of Fe0 specimens in the same layer. Similarly, the chloride concentrations values of DCf1, DCf2, DCf3 and DCf4 specimens are reduced by 14.23%, 28.93%, 23.43% and −4.90% compared with those of Ff0 specimens in the same layer as shown in Figure 3c. As shown in Figure 3, the chloride concentration values of DCd2, DCe2 and DCf2 specimens are average respectively reduced by 35.04%, 30.21% and 22.23% compared with those of Fd0, Fe0 and Ff0 specimens. The chloride concentration values of DCd3, DCe3 and DCf3 specimens are average respectively 1.20 times, 1.17 times and 1.13 times higher than those of DCd2, DCe2 and DCf2 specimens with the increase of sustained compressive stress. In the same way, the chloride concentration of DCd4, DCe4 and DCf4 specimens are significantly higher than those of DCd3, DCe3 and DCf3. From the above test data, it can be seen that the concrete with high *w/c* ratio was more susceptible to external sustained compressive stress, and the chloride diffusion performance of C45 concretes were higher than those of C55 concretes. When the applied sustained compressive stress is less than or equal to 0.3·*f_c_*, the chloride concentration concrete specimens decreases, and the diffusion of chloride ion was hindered. The chloride ion diffusion of concrete specimens would be promoted while the applied sustained compressive stress was greater than 0.3·*f_c_*.

**Figure 4:**
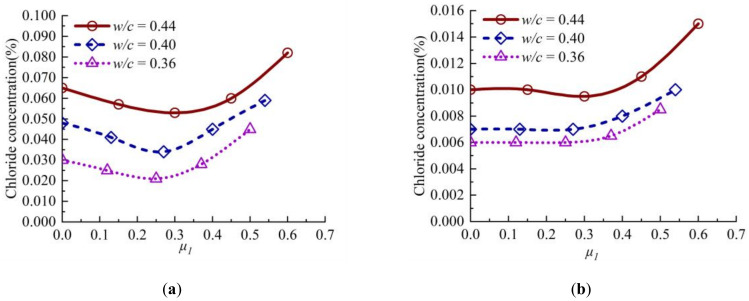
Variation curve of chloride concentration of concrete with stress level *μ_1_* at different depths:(**a**) 12.5 mm; (**b**) 22.5 mm.

The variation curves of chloride concentration with stress level *μ_1_* at the diffusion depth of 12.5 mm and 22.5 mm in concrete are shown in Figure 4. It can be seen from Figure 4a that with the increase of stress level, the chloride concentration in concrete decreases slightly first and then increases rapidly. When the values of *μ_1_* is close to 0.3, the chloride concentration in concrete with different water/cement ratio decreases. The chloride concentration in concrete remains unchanged as shown in Figure 4b. The influence of compressive stress on chloride concentration with large depth when the values of *μ_1_* is less than or equal to 0.3. Shown in Figure 4a,b, the chloride concentration in concrete increases when *μ_1_* is greater than 0.3. Under the same sustained compressive stress, the chloride concentration of C45 concrete specimen is higher than that of C55 concrete specimen.

### 3.2. Distribution of Chloride Concentration under Different Lateral Compressive Stresses

**Figure 5:**
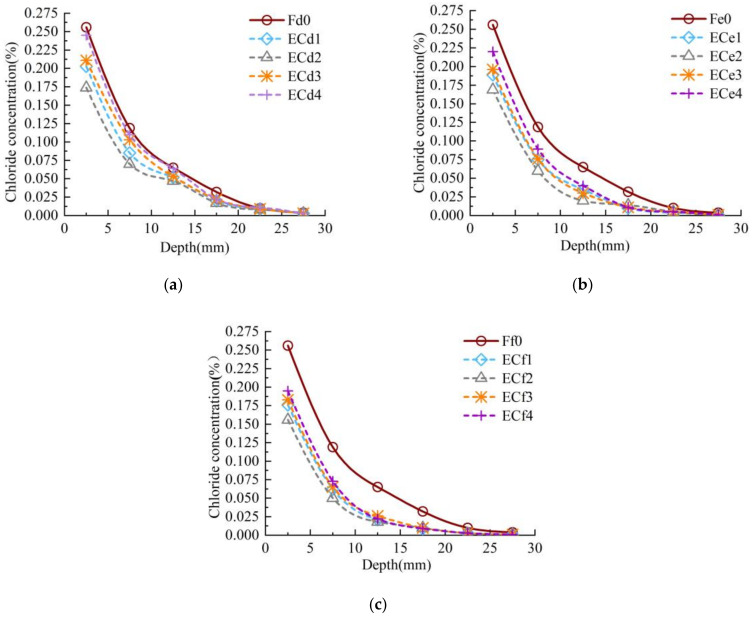
Chloride concentration distribution of concrete with three *w/c* ratios under biaxial sustained stress: (**a**) *w/c* = 0.44; (**b**) *w/c* = 0.4; (**c**) *w/c* = 0.36.

The chloride concentration distribution of concrete under biaxial sustained compressive stress (i.e., Categories E) and zero stress (i.e., Categories F), as shown in Figure 5. Take the chloride concentration values at a depth of 7.5mm as an example. The chloride concentration values of ECd1, ECd2, ECd3 and ECd4 specimens are 28.57%, 41.24%, 30.15% and 7.63% lower than those of Fd0 specimens in the same layer as seen in Figure 5a. It can be seen from Figure 5b that the chloride concentrations values of ECe1, ECe2, ECe3 and ECe4 specimens are 25.72%, 38.56%, 25.15% and 11.87% lower than those of Fe0 specimens in the same layer. Similarly, the chloride concentrations values of ECf1, ECf2, ECf3 and ECf4 specimens are reduced by 21.42%, 34.43%, 23.52% and 14.13% compared with those of Ff0 specimens in the same layer as shown in Figure 5c. Then, the chloride concentrations of ECd2, ECe2 and ECf2 specimens are respectively reduced by 50.03%, 45.22% and 41.54% compared with those of Fd0, Fe0 and Ff0 specimens. However, the chloride concentration of ECd3, ECe3 and ECf3 specimens are significantly 1.21 times, 1.16 times and 1.13 times higher than those of ECd2, ECe2 and ECf2 specimens with the increase of lateral sustained compressive tress. The experimental results show that the concretes with high *w/c* keeps good chloride ion diffusion performance even under biaxial sustained compressive stress. When the axial sustained compressive stress was about 0.3·*f_c_* and the lateral compressive stress was close to 0.15·*f_c_*, the chloride concentration of concrete decreases more obviously than those of under the uniaxial sustained compressive stress was about 0.3·*f_c_*. The diffusion ability of chloride ions in concrete increases while the axial sustained compressive stress was constant and the lateral sustained compressive stress was greater than 0.15*f_c_*. The increase was not significant.

**Figure 6:**
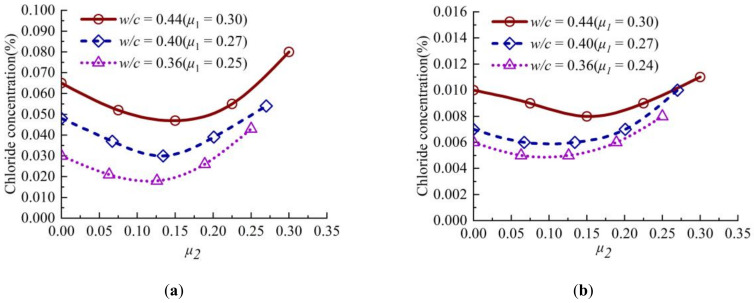
Variation curve of chloride concentration of concrete with stress level *μ_2_* at different depths: (**a**) 12.5 mm; (**b**) 22.5 mm.

The variation curves of chloride concentration with stress level *μ*_2_ at the diffusion depth of 12.5 mm and 22.5 mm in concrete are shown in Figure 6. It can be seen from Figure 6 that the chloride concentration in concrete first decreases slightly, and then increases rapidly with the increase of stress level. When the lateral stress level *μ*_2_ is less than or equal to 0.15, the chloride concentration in concrete decreases, and when *μ*_2_ is greater than 0.15, the chloride concentration in concrete increases greatly. Compared with Figure 4, it could be seen from the chloride concentration with an erosion depth of 22.5 mm that biaxial sustained compressive stress has an impact on the chloride ion concentration in the deep depth.

## 4. Chloride Diffusion Coefficient under Different Stress States

The chloride ions diffuse from the concrete surface with high chloride concentration to the concrete interior with low concentration chloride when concrete is exposed to chloride [24]. The diffusion process can be described by Fick’s second diffusion law, as given by Equation (2).

(2) $$\frac{\partial C(x,t)}{\partial t}=D_{c}(\frac{\partial^{2}C(x,t)}{\partial x^{2}})$$

where *C(x, t)* is the chloride concentration at the erosion depth of *x* at the exposure time *t*. *D_c_* is the chloride diffusion coefficient, which is a to-be-solved quantity. *t* is the time of exposure to chlorine salt.

Theoretically, Equation (2) is a partial differential equation with no analytical solution. It can be obtained by assuming that the diffusion of chloride ions in concrete is one-dimensional semi-infinite medium, and the surface chloride concentration is constant. As if the initial chloride concentration in concrete is zero, an error function solution of Equation (3) [25] can be got. Equation (3) can be obtained by sorting out Equation (2).

(3) $$C(x,t)=C_{s}[1-erf(\frac{x}{2\sqrt{D_{c}t}})]$$

where *C_s_* is the chloride concentration on the concrete surface. $erf(\frac{x}{2\sqrt{D_{c}t}})$ is the error function, which can be expressed by the following Equation.

(4) $$erf(\frac{x}{2\sqrt{D_{c}t}})=\frac{2}{\sqrt{\pi }}\int_{0}^{\frac{x}{2\sqrt{D_{c}t}}}e^{-\eta^{2}}d\eta$$

Combined with the chloride concentration in Figure 3 and Figure 5, the apparent chloride diffusion coefficient is solved by Equations (3) and (4). The solution results are shown in Table 5 and Table 6.

It can be seen from Table 5 and Table 6 that there is a functional relationship between chloride diffusion coefficient and stress level. Here, the stress function *f* is introduced to represent the relationship between chloride diffusion coefficient and stress level, as.

(5) $$f=\frac{D_{c(}{}_{ij)}}{D_{c(i0)}}$$

where *D_c(i0)_* indicates chloride diffusion coefficient under unstressed condition, and *i* refers to concrete specimens with different *w/c* ratios, with *i* = 1, 2 and 3. *D_c(ij)_* indicates chloride diffusion coefficient under different compressive stress states, and *j* refers to four kinds of compressive stress states, with *j* = 1, 2, 3 and 4. Under uniaxial or biaxial compression cases, *f* is expressed as *f(μ_1_)* or *f(μ_1_, μ_2_),* respectively.

### 4.1. Stress Level Function under Uniaxial Sustained Stress

**Figure 7:**
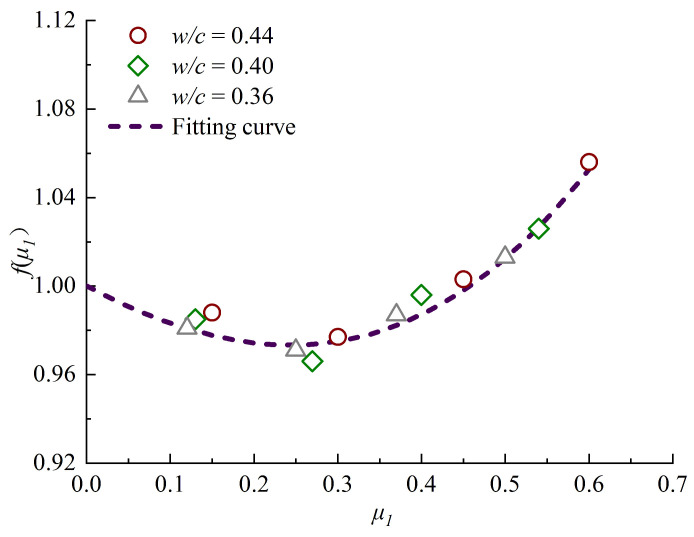
Stress function versus stress level *μ_1_*.

The relationship between stress level *μ_1_* and stress function *f(μ_1_)* is shown in Figure 7, which is approximately a cubic curve.

The following equation is regressed from the test data with a correlation coefficient R^2^ of 0.95, and the value of *p* is less than 0.0001, so the value of *μ_1_* has a significant influence on the value of *f(μ_1_)*.

(6) $$f(\mu_{1})=1-0.2\mu_{1}+0.3\mu_{1}^{2}+0.3\mu_{1}^{3}$$

The apparent chloride diffusion coefficient model under uniaxial sustained stress level can be obtained as.

(7) $$D(\mu_{1})=D_{0}(1-0.2\mu_{1}+0.3\mu_{1}^{2}+0.3\mu_{1}^{3})$$

**Figure 8:**
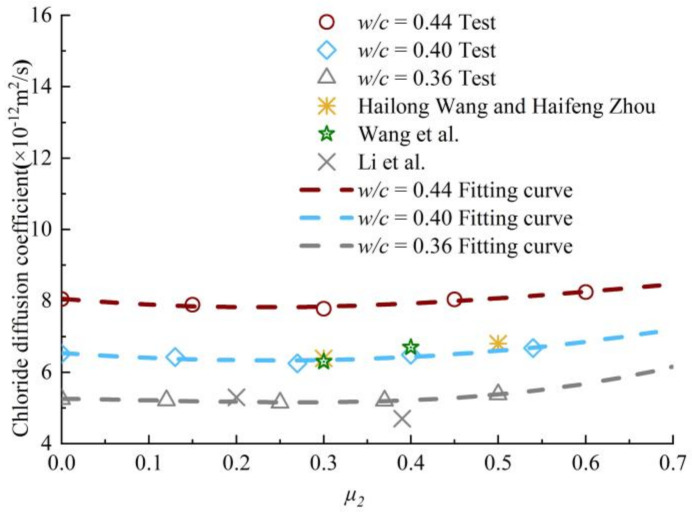
Experimental determination of chloride diffusion coefficient under uniaxial sustained compressive stress.

The rationality of the derived apparent diffusion coefficient model is verified by the applicability of the model using the experimental data in Yuanzhan Wang and Haifeng Zhou [26], Wang et al. [27] and Li et al. [28]. Among them, the *w/c* ratio of concrete specimens is 0.38 used by Yuanzhan Wang and Haifeng Zhou [26]. Two types of compressive stress levels are 0.3 and 0.5. With respect to Wang et al. [27], the *w/c* ratio of concrete specimens is 0.39. The compressive stress levels applied to the two groups of specimens is respectively 0.3 and 0.4. The *w/c* ratio of concrete specimens used by Li et al. [28] is 0.36. The compressive stress levels applied to the two groups of specimens are 0.2 and 0.39, respectively. Except for the case of Li et al. [28] *μ_1_* = 0.4, the experimental data of other researchers are close to the calculated values of the model, as shown in Figure 8. Therefore, the diffusion model derived in this paper can be used to predict the state of concrete with *w/c* ratio of 0.44–0.36 under uniaxial sustained compressive stress.

### 4.2. Stress Level Function under Biaxial Sustained Compressive Stress

**Figure 9:**
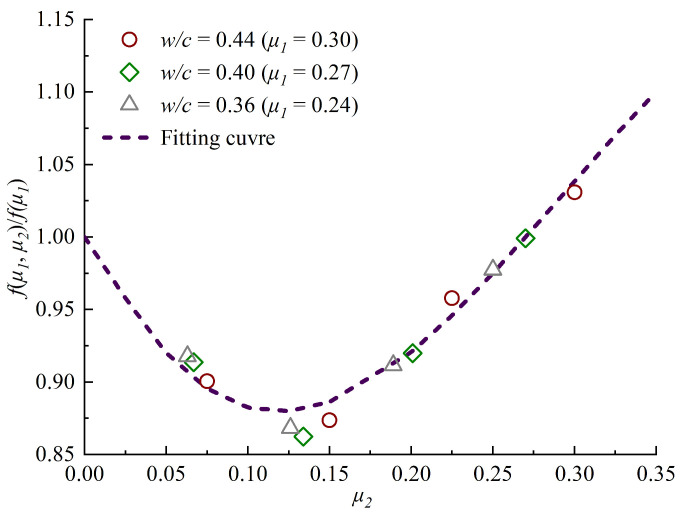
Relationship between *f(μ_1_, μ_2_)/f(μ_1_)* and *μ_2_.*

The chloride ions diffusion capacity of concrete is affected by *μ_1_* and *μ_2_* at the same time under biaxial compressive load. The chloride diffusion coefficient varies with the ratio of *μ_1_* to *μ_2_* [29]. Therefore, the relationship between *f(μ_1_, μ_2_)*/*f(μ_1_)* and *μ_2_* should be established. To determine the relationship between the surface chloride accumulation and the lateral sustained compressive stress level, *f(μ_1_, μ_2_)*/*f(μ_1_)* is plotted against the lateral sustained compressive stress level *μ_2_* in Figure 9.

According to the regression of experimental data, the following equation is obtained and the correlation coefficient R^2^ is 0.96, and the value of *p* is less than 0.0001, so the value of *μ_2_* has a significant influence on the value of *f(μ_1_, μ_2_)*/*f(μ_1_)*. Then, the biaxial sustained stress influencing equation is

(8) $$f(\mu_{1},\mu_{2})/f(\mu_{1})=1.01-2.39u_{2}+12.59u_{2}^{2}-14.36u_{2}^{3}(0<u_{2}\leq 0.3)$$

According to Equation (8), the apparent chloride diffusion coefficient model under biaxial sustained compressive stress is obtained as

(9) $$D(\mu_{1},\mu_{2})=D_{0}(1.01-2.39\mu_{2}+12.59\mu_{2}^{2}-14.36\mu_{2}^{3})(1-0.2\mu_{1}+0.3\mu_{1}^{2}+0.3\mu_{1}^{3})$$

**Figure 10:**
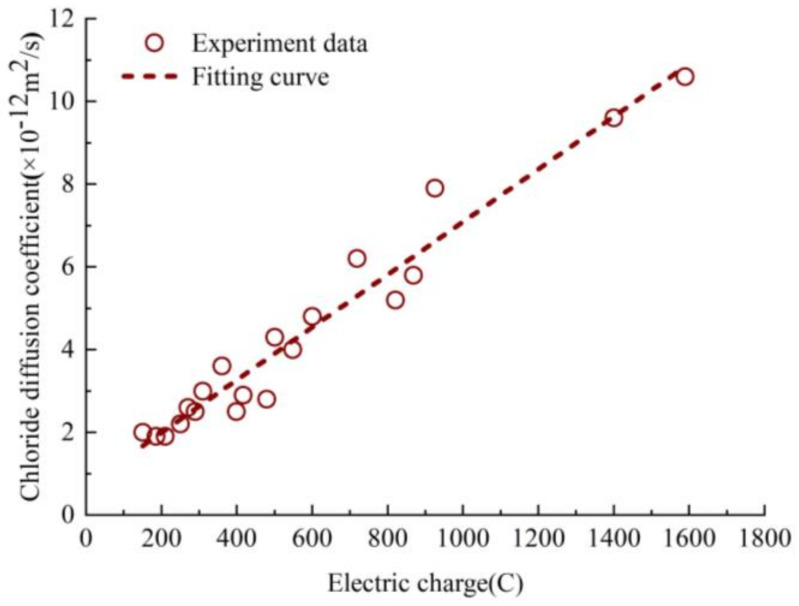
The Variation of chloride ion diffusion coefficient with charge.

At present, little research on chloride ions diffusion test of ordinary concrete under biaxial sustained compressive stress. The chloride diffusion performance of concrete containing admixture under biaxial stress is studied in Shumei Wang [29] and Xingcai Wei [15]. The diffusion law of chloride ion is described by the charge. Both charge and chloride diffusion coefficient can describe the migration law of chloride in concrete [30]. Figure 10 shows the variation of experimental data of chloride diffusion coefficient with the electric charge [31,32]. 

The relationship between chloride diffusion coefficient and charge can be described by a linear function, and the correlation coefficient R^2^ tends to be 1, and the value of *p* is less than 0.0001, so the value of electric charge has a significant influence on the value of chloride diffusion coefficient, as seen in Equation (10).

(10) D’=0.006C+0.721

where *D’* is the apparent chloride diffusion coefficient. *C* is the electric charge in concrete. 

**Figure 11:**
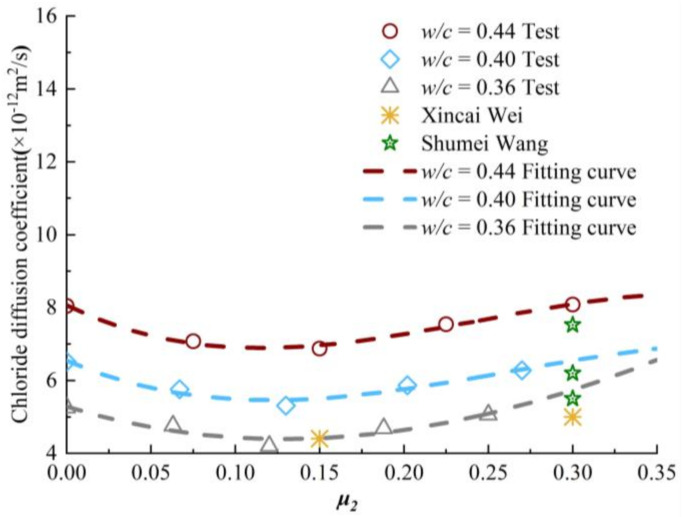
Verification of chloride diffusion coefficient under biaxial sustained compressive stress.

In this section, the applicability of chloride ion diffusion coefficient model is verified by the experiment data in Shumei Wang [29] and Xingcai Wei [15]. Among them, Shumei Wang [29] applied the biaxial compressive stress level of *μ_1_* = *μ_2_* = 0.3, and the *w/c* ratio of concrete specimens are 0.42, 0.4 and 0.38. With respect to Xingcai Wei [15], the *w/c* ratio of concrete specimens is 0.32. The applied axial compressive stress is 0.3, and the applied lateral compressive stresses are 0.3 and 0.15, respectively. The above literatures describe the diffusion performance of chloride ions in concrete by electric charge. It is necessary to convert the charge into the chloride diffusion coefficient by using Equation (9). Figure 11 shows the variation of chloride diffusion coefficient with μ_2_ in the range of *μ_1_* = 0.27~0.3.

As shown in Figure 11, the model value of chloride diffusion coefficient of concrete with *w/c* ratio of 0.32 is quite different from the experimental value when the axial and lateral compressive stresses are both 0.3·*f_c_*. However, the model value of chloride diffusion coefficient of concrete with *w/c* ratio of 0.32 is almost equal to the experimental value, when the axial and lateral sustained compressive stress levels are 0.3·*f_c_* and 0.15·*f_c_*, respectively. The model calculation values of chloride diffusion coefficient in concrete with *w/c* ratio of 0.42, 0.4 and 0.38 are corresponded very well with the experimental values. Therefore, the diffusion model derived in this paper can be used to predict the situation of the biaxial compressive stress is less than or equal to 0.3·*f_c_* and the *w/c* ratio of 0.44-0.36.

## 5. Conclusions and Comments

To characterize the diffusivity of concrete under sustained compressive stress, the systematic experiments and numerical studies are carried out on ordinary concrete exposed to one-dimensional chlorine salt erosion under different sustained compressive stress conditions. This paper introduces the experimental study on the influence of different sustained compressive stresses on chloride ion diffusivity of concrete in chloride ion solution, and the influence of water/cement ratio on chloride ion diffusivity. The chloride concentration distribution of concrete under uniaxial and biaxial continuous compressive stress was measured to evaluate the chloride ion diffusion characteristics of concrete materials, and the chloride ion diffusion coefficient was calculated to test the influence of different sustained compressive stress states on the chloride ion diffusion process. Finally, chloride diffusion models under different stress states are derived by using chloride diffusion coefficient. According to the experimental results, the following conclusions are drawn.

1. The existence of sustained compressive stress changes the diffusion process of chloride ions from environmental solution into concrete. It is found that the maximum chloride concentration of concrete specimens under axial sustained compressive stress of 0.3·*f_c_* are about 35% lower than that those of under no stress. The highest concentration chloride in the concretes with the axial and lateral sustained compressive stress of 0.3·*f_c_* and 0.15·*f_c_* are respectively reduced by about 50% compared with those of without stress. In addition, the diffusion process and concentration of chloride ions will increase when the sustained compressive stress is higher than 0.3*f_c_*. For biaxial concrete with constant axial sustained compressive stress, the chloride concentration distribution with lateral sustained compressive stress greater than 0.15·*f_c_* is 1.27-1.1 times that of lateral sustained compressive stress. With the increase of sustained compressive stress, the diffusion distribution of chloride ions will be further enhanced.
2. Under the same sustained compressive stress, the chloride concentration of C45 concrete specimens is higher than that of C55 concrete specimen. This is used for the higher *w/c* ratio produces lower grade concrete will leads to the concrete structure is not dense and is more susceptible to the influence of sustained compressive stress. Therefore, concrete specimens with higher *w/c* ratio have higher permeability. The chloride diffusion coefficient model, considering the effect of uniaxial and biaxial sustained compressive stress of concrete, is established. The model is helpful to predict the durability of concrete structures in chlorine-laden environment.
3. It is recommended that further research to measure the cracks in concrete with sustained stress states during the experiment. The pore size distribution of concrete under sustained stress and crack depth, so as to expand and improve the model potentially.

## Figures and Tables

**Table 1:** Chemical composition of cement.

Ingredients	SiO_2_	Al_2_O_3_	CaO	MgO	SO_2_	Loss on Ignition
Mass Percent (%)	22.45	6.64	63.31	2.63	2.38	1.89

**Table 2:** Mix ratio of concrete test block.

Type	Unit Content (kg/m^3^)					
	*w/c*	Water	Cement	Fine Aggregate	Coarse Aggregate	SP
C45	0.44	180	409.09	543.27	1267.64	3.40
C50	0.40	180	450.00	531.00	1239.00	3.15
C55	0.36	180	500.00	516.00	1204.00	3

**Table 3:** Test specimens under uniaxial stress.

Specimen	*w/c*	*f_c_* (MPa)	*σ_cx_* (MPa)
DCd1	0.44	47.21	7.08(0.15*fc*)
DCd2	0.44	47.21	14.16(0.30*fc*)
DCd3	0.44	47.21	21.24(0.45*fc*)
DCd4	0.44	47.21	28.33(0.60*fc*)
DCe1	0.40	52.68	7.08(0.13*fc*)
DCe2	0.40	52.68	14.16(0.27*fc*)
DCe3	0.40	52.68	21.24(0.40*fc*)
DCe4	0.40	52.68	28.33(0.54*fc*)
DCf1	0.36	56.78	7.08(0.12*fc*)
DCf2	0.36	56.78	14.16(0.25*fc*)
DCf3	0.36	56.78	21.24(0.37*fc*)
DCf4	0.36	56.78	28.33(0.50*fc*)

**Table 4:** Test specimens under biaxial stress.

Specimen	*w/c*	*σ_cx_*/*σ_cy_*	*f_c_* (MPa)	*σ_cx_* (MPa)	*σ_cy_* (MPa)
ECd1	0.44	1:0.25	47.21	14.16(0.30*fc*)	3.54(0.075*fc*)
ECd2	0.44	1:0.5	47.21	14.16(0.30*fc*)	7.08(0.150*fc*)
ECd3	0.44	1:0.75	47.21	14.16(0.30*fc*)	10.63(0.225*fc*)
ECd4	0.44	1:1	47.21	14.16(0.30*fc*)	14.16(0.300*fc*)
ECe1	0.40	1:0.25	52.68	14.16(0.27*fc*)	3.54(0.067*fc*)
ECe2	0.40	1:0.5	52.68	14.16(0.27*fc*)	7.08(0.134*fc*)
ECe3	0.40	1:0.75	52.68	14.16(0.27*fc*)	10.63(0.201*fc*)
ECe4	0.40	1:1	52.68	14.16(0.27*fc*)	14.16(0.270*fc*)
ECf1	0.36	1:0.25	56.78	14.16(0.25*fc*)	3.54(0.063*fc*)
ECf2	0.36	1:0.5	56.78	14.16(0.25*fc*)	7.08(0.126*fc*)
ECf3	0.36	1:0.75	56.78	14.16(0.25*fc*)	10.63(0.189*fc*)
ECf4	0.36	1:1	56.78	14.16(0.25*fc*)	14.16(0.250*fc*)

**Table 5:** Chloride diffusion coefficient under sustained uniaxial stress.

Specimen	*D_c_* (×10^−12^ m^2^/s)	Specimen	*D_c_* (×10^−12^ m^2^/s)	Specimen	*D_c_* (×10^−12^ m^2^/s)
Fd0	8.05	Fe0	6.52	Ff0	5.25
DCd1	7.95	DCe1	6.42	DCf1	5.15
DCd2	7.86	DCe2	6.30	DCf2	5.10
DCd3	8.07	DCe3	6.49	DCf3	5.18
DCd4	8.50	DCe4	6.69	DCf4	5.32

**Table 6:** Chloride diffusion coefficient under sustained biaxial stress.

Specimen	*Dc* (×10^−12^ m^2^/s)	Specimen	*Dc* (×10^−12^ m^2^/s)	Specimen	*Dc* (×10^−12^ m^2^/s)
Fd0	8.05	Fe0	6.52	Ff0	5.25
ECd1	7.08	ECe1	5.73	ECf1	4.70
ECd2	6.87	ECe2	5.41	ECf2	4.43
ECd3	7.54	ECe3	5.81	ECf3	4.61
ECd4	8.08	ECe4	6.28	ECf4	5.00
